# Antioxidant Nutrients and Diabetes and Its Complications: A Narrative Review on the Roles of Vitamin E, Vitamin C, and Selenium

**DOI:** 10.7759/cureus.95075

**Published:** 2025-10-21

**Authors:** Mahesh Kumar Mummadi, Shankar Sapavat, Indiramma Ettey, Tejaswini Appala, Jag Jeevan Babu Geddam

**Affiliations:** 1 Clinical Epidemiology, Indian Council of Medical Research (ICMR) National Institute of Nutrition, Hyderabad, IND; 2 Public Health Nutrition, Indian Council of Medical Research (ICMR) National Institute of Nutrition, Hyderabad, IND

**Keywords:** antioxidant, diabetic neuropathy, oxidative stress, selenium, vitamin e

## Abstract

This narrative review summarizes the evidence on vitamin E, vitamin C, and selenium in preventing or mitigating diabetes, diabetic neuropathy, and associated complications. A comprehensive literature search was conducted in MEDLINE for English language studies from January 2000 to April 2025 using terms such as “vitamin E,” “selenium”, “antioxidant”, “diabetes”, and “neuropathy”. Randomized clinical trials were included without grading restrictions. Antioxidants such as vitamin E, selenium, and vitamin C show potential in reducing oxidative stress, improving glycemic control, and alleviating diabetic complications. However, findings are inconsistent, emphasizing the need for personalized approaches and further trials to clarify their therapeutic roles. A balanced diet rich in antioxidant-containing foods continues to be recommended to support metabolic health and reduce the risk of diabetes-related complications.

## Introduction and background

According to the WHO (World Health Organization) [1], the number of people living with diabetes has seen a significant rise, increasing from 200 million in 1990 to 830 million in 2022. This surge has been especially pronounced in low- and middle-income countries, where the growth rate has outpaced that of high-income nations. More than half of adults with diabetes in 2022 did not receive medication, with treatment coverage lowest in low- and middle-income settings. Globally, 14% of adults were living with diabetes in 2022, up from 7% in 1990. Despite this increase, more than half of individuals over the age of 30 with diabetes were not receiving medication. Treatment coverage remains notably low in low- and middle-income countries.

In 2021, diabetes was directly responsible for 1.6 million deaths, with nearly half (47%) of those deaths occurring before the age of 70. Furthermore, 530,000 deaths were linked to kidney disease, and 11% of cardiovascular deaths were associated with diabetes. Since 2000, diabetes-related mortality rates have risen, while the global risk of dying from major noncommunicable diseases has generally decreased [1].

Diabetes is a long-term health condition in which the pancreas either fails to produce enough insulin or the body is unable to use the insulin effectively. Insulin plays a key role in managing blood sugar levels, and without proper control, diabetes can result in high blood sugar (hyperglycemia), which may harm various organs, especially the nerves and blood vessels.

There are three primary types of diabetes: type 1, type 2, and gestational diabetes mellitus (GDM). Type 1 diabetes is caused by an autoimmune response that destroys the insulin-producing beta cells in the pancreas, resulting in an absolute lack of insulin. Type 2 diabetes is marked by both insulin resistance and impaired beta-cell function, leading to insufficient insulin production. GDM develops during pregnancy due to temporary glucose intolerance and usually resolves after delivery. Other less common forms include maturity-onset diabetes of the young (MODY), neonatal diabetes, type 3c diabetes, latent autoimmune diabetes in adults (LADA), cystic fibrosis-related diabetes, and diabetes caused by steroid use [2].

Diabetic individuals struggle with glucose metabolism and fatty acid synthesis due to insufficient insulin secretion or impaired insulin action. This disrupts normal metabolic processes, increasing the risk of vascular complications, including cardiovascular disease, retinopathy, nephropathy, and neuropathy. Elevated glucose levels, hypertension, obesity, and oxidative stress are key contributors to these complications.

The management of type 2 diabetes mellitus typically includes medications such as metformin, along with regular physical activity. The Diabetes Control and Complications Trial (DCCT) highlighted the critical role of maintaining tight blood glucose control in minimizing the risk of complications. One of the key contributors to these complications is oxidative stress, which results from the production of free radicals due to elevated blood sugar levels. As a result, antioxidant therapy is being explored as a promising approach to address these effects [2].

Diabetic neuropathy (DN) is a prevalent but not fully understood complication of diabetes, affecting over half of those with long-standing diabetes. It is the primary cause of diabetes-related hospitalizations and non-traumatic limb amputations. While DN typically develops later in individuals with type 1 diabetes, it often emerges earlier in those with type 2 diabetes, though the exact reasons for this difference are still uncertain. Oxidative stress - caused by excessive free radical production or weakened antioxidant defenses - is believed to play a central role in the onset and progression of DN. This condition encompasses a range of nerve disorders, impacting different parts of the nervous system and resulting in a variety of clinical symptoms [3].

Oxidative stress, primarily induced by hyperglycemia and the subsequent generation of free radicals, plays a pivotal role in the onset and progression of complications associated with diabetes. This phenomenon arises from an imbalance between the levels of oxidants and antioxidants in the body. Under conditions of uncontrolled diabetes, reactive oxygen species (ROS) are consistently generated from multiple cellular sources, including hyperglycemia itself. ROS includes a variety of free radicals involved, including superoxide anion (O2−), hydroxyl radical (OH), alkoxyl radical (RO), peroxyl radical (ROO), hydrogen peroxide (H2O2), and singlet oxygen (O2) [4].

In a healthy system, the cellular antioxidant defense mechanisms, comprising both enzymatic and non-enzymatic antioxidants, maintain the natural balance of ROS to prevent cellular damage. However, when ROS generation exceeds the capacity of these defense systems, excess ROS can damage essential biomacromolecules such as DNA, proteins, and lipids. This damage can trigger a cascade of detrimental effects, including the disruption of protein pathways that contribute to age-related muscle wasting. Furthermore, the heightened oxidative stress plays a crucial role in the development of several chronic complications commonly seen in diabetes, cardiovascular disease, nephropathy, neuropathy, and retinopathy [5]. The co-occurrence of infections such as COVID-19 with underlying chronic conditions, particularly diabetes, markedly intensifies disease severity, complicates clinical management, and increases the risk of adverse outcomes [6].

Antioxidants are essential for protecting cells from oxidative stress, which arises when there is an imbalance between ROS and the body’s capacity to counteract them. These protective compounds can be generated within the body or acquired from external sources such as food. Antioxidants are generally classified into two main types: enzymatic and non-enzymatic.

Enzymatic antioxidants include superoxide dismutase (SOD), catalase (CAT), glutathione S-transferase (GST), and glutathione peroxidase (GPx). SOD converts superoxide anions (O2−) into H2O2, which is then further neutralized by CAT and GPx into water, thereby mitigating potential damage to cellular structures. GST plays an essential role in detoxifying reactive electrophilic species by facilitating their conversion into more hydrophilic forms through conjugation with reduced glutathione (GSH), which makes them easier to excrete.

Non-enzymatic antioxidants include GSH, uric acid, carotenoids, flavonoids, lipoic acid, and vitamins A, C, and E. These compounds also serve critical functions in protecting cells from oxidative damage. For example, vitamins C and E, along with lipoic acid, help terminate lipid peroxidation processes, which prevent cellular membrane damage. Additionally, flavonoids have been shown to scavenge free radicals, further contributing to the body’s defense mechanisms.

In addition to these well-known antioxidants, specialized proteins such as peroxiredoxins, thioredoxins, and guardians also function as antioxidants, providing an added layer of protection against oxidative stress [7-10].

Aim and objectives of this review

The aim of the review is to evaluate the effects of vitamin E, selenium, and other antioxidant supplementation on oxidative stress, inflammation, and glycemic control in patients suffering from diabetes and its complications. The objectives are to determine the role of antioxidants in improving glycemic control in patients suffering from diabetes and cardiometabolic diseases and to estimate the changes in oxidative stress and inflammatory markers in patients suffering with diabetes and its complications after antioxidant supplementation.

## Review

Search methodology

Sources and Selection Criteria

The present work adopts a narrative review approach to synthesize current evidence on the role of antioxidant nutrients in diabetes mellitus and its associated vascular complications. We conducted a comprehensive literature search using major biomedical database MEDLINE (via PubMed) to ensure broad coverage and high-quality peer-reviewed sources. The search period was restricted from January 2000 to April 2025, capturing developments over a 25-year period in antioxidant-based interventions related to diabetes management.

**Figure 1:**
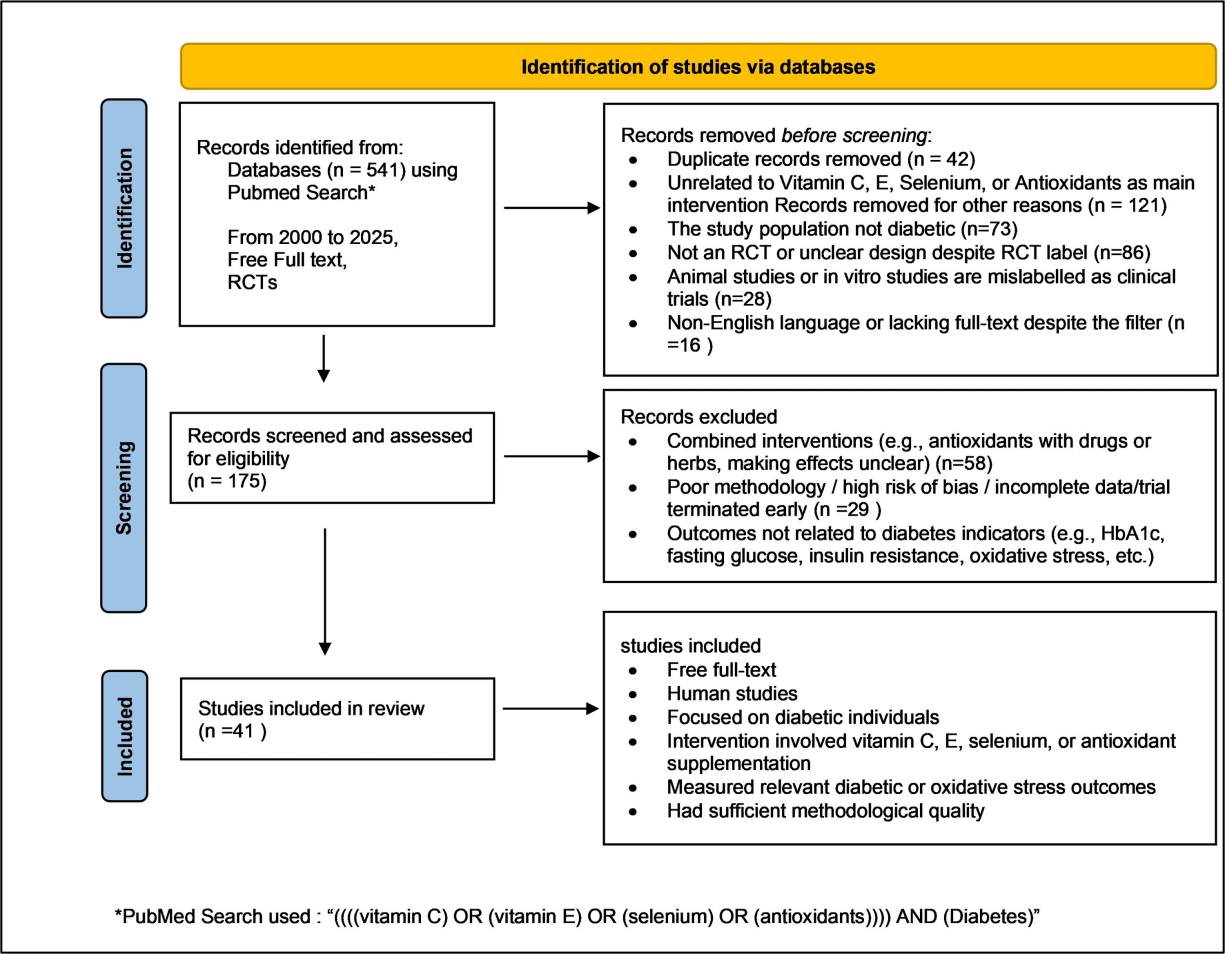
PRISMA flow diagram for study selection on antioxidant supplementation in diabetes The diagram shows the selection process of studies from PubMed (2000–2025). From 541 records, 41 randomized controlled trials were included after excluding duplicates, non-relevant, poor-quality, or non-diabetes studies, focusing on antioxidant interventions with relevant diabetic outcomes. PRISMA, Preferred Reporting Items for Systematic Reviews and Meta-Analyses

The search strategy was carefully developed by combining controlled vocabulary (such as Medical Subject Headings [MeSH] in PubMed) and free-text keywords to enhance the sensitivity and specificity of the retrieval. The following key terms and their various permutations were used in isolation or combination: “vitamin E”, “selenium”, “vitamin C”, “antioxidants”, “oxidative stress”, “diabetes mellitus”, “diabetic complications”. Boolean operators such as AND/OR were used to narrow or broaden the search criteria depending on the scope of each term. Although this is a narrative review, we used a systematic search strategy (Figure 1) adapted from PRISMA guidelines [11]. 

Articles were initially screened based on titles and abstracts and subsequently selected through full-text evaluation based on relevance to the review objectives. To maintain scientific rigor and contextual applicability, inclusion criteria were limited to English-language publications reporting on mechanistic insights, clinical outcomes, or biochemical pathways involving antioxidant nutrients in human populations that simulate diabetic pathophysiology. Studies focusing on isolated nutrient supplementation as well as combinations of antioxidants were included, provided that they addressed oxidative stress or vascular parameters linked to diabetic conditions.

While an attempt was made to ensure methodological diversity and global representation, the final selection of articles primarily included those with a biomedical, biochemical, or clinical focus. Review articles, editorials, conference abstracts, and grey literature were excluded. In total, the final set of included studies reflects clinical trials that comprehensively address the role of antioxidants in diabetes and its vascular sequelae.

Due to the considerable heterogeneity in study designs, dosage protocols, outcome measures, antioxidant combinations, duration of interventions, and subject characteristics, it was not feasible to conduct a quantitative meta-analysis. Additionally, the variation in reporting standards and methodological quality across studies rendered formal risk-of-bias assessments impractical. Instead, the current narrative synthesis aims to present an integrative view of the available evidence, highlighting recurring patterns, inconsistencies, and areas requiring further research, without pooling data statistically.

This methodology was chosen to preserve the contextual richness and complexity of findings in this evolving field, particularly where interventions target oxidative stress-mediated pathways - a key underlying mechanism in the progression of diabetic complications especially neuropathy.

Oxidative stress and antioxidants

Oxidative Stress

Oxidative stress arises when the production of reactive molecules, such as ROS and reactive nitrogen species (RNS), exceeds the body’s capacity to neutralize them. ROS includes both free radicals such as superoxide (O2−) and hydroxyl (OH), as well as non-radicals such as H2O2. RNS comprises free radicals such as nitric oxide (NO) and nitrogen dioxide (NO2−), along with non-radical species such as proximities (ONOO−) and nitrous acid (HNO2). While molecules such as O2−, NO, and ONOO− play important roles in normal physiological functions, their excessive accumulation can lead to harmful effects, including the development of cardiovascular complications in diabetes [12].

NO and Vascular Health

NO, produced from L-arginine by endothelial nitric oxide synthase (eNOS), plays a pivotal role in maintaining vascular health. It induces vasorelaxation by stimulating guanylate cyclase in vascular smooth muscle cells, resulting in the dilation of blood vessels. NO also inhibits platelet aggregation, reduces leukocyte adhesion, and exerts antiproliferative effects on smooth muscle cells. However, when NO interacts with superoxide (O2-), it forms proximities (ONOO-), a reactive molecule that can cause cellular damage and inflammation. The outcome of NO's activity - whether protective or harmful - depends largely on the availability of O2- and the balance between these reactive molecules [13].

Interaction Between ROS and RNS

**Figure 2:**
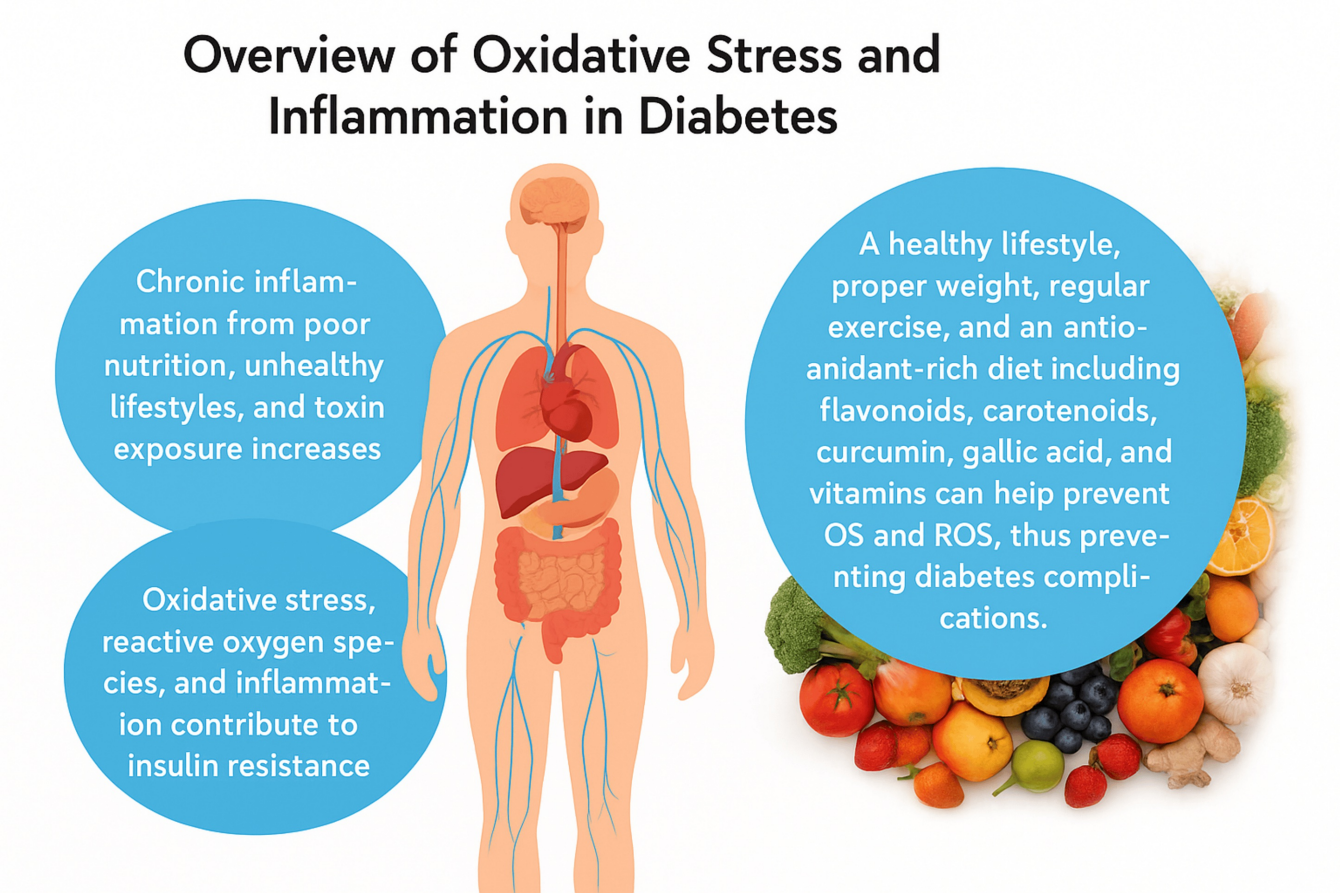
Overview of oxidative stress and inflammation in diabetes The figure illustrates how poor nutrition, unhealthy lifestyles, and toxin exposure contribute to chronic inflammation and oxidative stress, leading to insulin resistance. It also highlights that a healthy lifestyle with proper weight management, exercise, and an antioxidant-rich diet can reduce oxidative stress and reactive oxygen species, thereby preventing diabetes-related complications. This figure has been redrawn based on the 2024 publication by Weinberg Sibony et al. [15], with individual modifications to avoid copyright.

ROS and RNS can influence each other's production through radical chain reactions, amplifying their harmful effects. For instance, O2- is generated by enzymes such as NAD(P)H oxidase and xanthine oxidase, and during mitochondrial oxidative phosphorylation. Under normal conditions, O2- is neutralized by antioxidant defense systems, where it is converted into H2O2, which is then reduced to water (H2O) and oxygen (O2). However, in the presence of metals such as iron or copper, H2O2 can undergo a Fenton reaction, leading to the formation of the highly reactive hydroxyl radical (OH). This radical can initiate further cellular damage, contributing to oxidative stress and the development of various diseases [14]. In today's world, chronic inflammation - often driven by poor dietary habits, sedentary lifestyles, and exposure to environmental toxins - is becoming more widespread and is recognized as a major risk factor for long-term illnesses. Research indicates that oxidative stress, ROS, and inflammation may contribute to the onset of insulin resistance and impaired β-cell function (Figure 2).

Antioxidants

Antioxidants such as vitamin E and selenium play a vital role in diabetes management by neutralizing free radicals and ROS, which helps lower oxidative stress levels. Oxidative stress results from an imbalance between the production of these reactive molecules and the body’s ability to eliminate them or repair the damage they cause. When unchecked, this imbalance can lead to cellular injury, promoting the onset and worsening of diabetes and its related complications.

Despite efforts to diagnose and intervene early, there is currently no effective global treatment for DN beyond strict blood glucose control. This is likely due to the insufficient understanding of its pathogenesis, the complex clinical presentations that may not reflect true disease progression, and the limitations in clinical trial designs. Furthermore, the development of potential treatments may not address the actual underlying causes. To overcome this challenge, it is crucial to thoroughly explore the specific mechanisms driving nerve fiber dysfunction and loss. This review highlights the latest advances in understanding the pathogenesis of DN and outlines promising directions for future treatment.

The duration of diabetes and glycated hemoglobin (HbA1c) levels are closely linked to an increased risk of neuropathy. The Diabetes Control and Complications Trials (DCCT) showed that intensive insulin treatment in type 1 diabetes reduced HbA1c by 2% (from 9% to 7%) over 6.5 years and lowered neuropathy incidence by 60%. These effects persisted even eight years after the trial, demonstrating "legacy effects" of tight glucose control. In type 2 diabetes, the Kumamoto study found that intensive insulin therapy for seven years improved nerve function. However, the UKPDS study showed that glucose control in type 2 diabetes did not significantly reduce neuropathy, despite lowering HbA1c by 0.9%. The EURO-Diab study indicated that blood glucose control, diabetes duration, hypertension, hyperlipidemia, and smoking were key risk factors for neuropathy in type 1 diabetes. Although hyperlipidemia was a risk factor in some studies, Japanese research did not find a significant link between lipid levels and neuropathy. Ultimately, high blood glucose leads to peripheral nerve damage through a metabolic cascade. Antioxidants are compounds that help protect the body by inhibiting oxidation, a chemical reaction that can produce free radicals. These free radicals can lead to cellular damage and have been linked to a range of health issues, including aging and chronic diseases. By neutralizing these harmful molecules, antioxidants play a key role in maintaining health and preventing oxidative stress [14].

Vascular supply of the peripheral nervous system

Peripheral Nerve Vasculature

The peripheral nerves have a sparse blood supply. Perineurium, the connective tissue surrounding the nerves, contains only a few arterioles that penetrate into the endoneurium (the innermost layer surrounding individual nerve fibers). This sparse vascular network leads to compromised blood flow and a lack of autoregulation, making peripheral nerves more susceptible to ischemia.

Endoneurial Microvessels and Leakage

Endoneurial microvessels, which supply nutrients to the nerves, are tightly connected by endothelial cells. However, in conditions such as diabetes, these vessels can become leaky, leading to a disruption in the integrity of the endoneurial tissue. This leakage, especially in the ganglion (a group of nerve cell bodies), affects the nutrient and oxygen supply to nerve terminals, which can cause further injury and dysfunction.

Autonomic Innervation and Diabetes

Autonomic nerve endings, which innervate the microvessels, play a role in regulating vascular tone. In diabetic conditions, these nerve endings are often lost, impairing the ability of the blood vessels to adjust their size and blood flow (vascular autoregulation). This further worsens the blood supply to the nerves, exacerbating the development of neuropathy. The vascular and structural organization of a peripheral nerve is shown by Yagihashi et al. [16].

Neuronal Architecture and Susceptibility

The long axons of neurons in the peripheral nervous system, covered by Schwann cells, are particularly vulnerable. Neuronal cell bodies are relatively small compared to the length of their axons, meaning the distal parts of these long axons are more prone to damage. These distal axons rely on the efficient transport of nutrients and nerve growth factors, but due to their long distance from the cell body, they are less able to support themselves and are more susceptible to damage, particularly under conditions like diabetes [17,18]. The vascular supply to the peripheral nervous system is limited, with few arterioles penetrating the endoneurium. Autonomic nerve endings interact with these arterioles, but peripheral nerves lack vascular autoregulation due to sparse innervation. In diabetes, the autonomic nerve endings are often lost, impairing the regulation of blood flow.

Pathological background of neuropathy

Peripheral Nerve Damage in Diabetes

DN primarily affects the distal and sensory nerve fibers, leading to their degeneration, axonal loss, and microangiopathy. This condition involves damage to the small blood vessels (microvessels) that supply the nerves, contributing to impaired nerve function. Both small and large nerve fibers are impacted in DN. Microvascular injury is believed to play a pivotal role in focal fiber loss, which, over time, aggregates into a more widespread loss of nerve fibers.

Microangiopathy and Vascular Injury

Microvascular damage, particularly in the endoneurial microvessels, which are the small blood vessels within the nerve fibers, is considered a key contributor to the development of DN. This vascular injury disrupts the delivery of oxygen and nutrients to the nerve tissue, leading to further nerve damage. However, the exact mechanisms remain a subject of debate. There are contradictions in understanding how microangiopathy fully explains the pathology of DN, particularly when considering factors such as prolonged hyperglycemia and the duration of diabetes, which may also play critical roles in nerve deterioration [19,20].

Peripheral Nerve Injury Due to Hyperglycemia

Hyperglycemia, or high blood sugar, can lead to peripheral nerve injury through a combination of metabolic changes and increased stress on the nerves [21]. Here's how this process generally works.

Elevated blood glucose levels: When blood glucose levels are persistently high, such as in uncontrolled diabetes, glucose begins to accumulate in the bloodstream. The body's cells, including nerves, are exposed to this excess glucose.

Increased production of sorbitol: One of the biochemical pathways affected by hyperglycemia is the polyol pathway, where excess glucose is converted into sorbitol. Sorbitol is a type of sugar alcohol that can accumulate within cells. Since sorbitol does not readily pass through the cell membrane, its accumulation inside nerve cells creates osmotic stress, which can lead to cellular swelling and damage over time.

Oxidative stress and inflammation: Persistent hyperglycemia results in elevated production of ROS, which are highly reactive molecules that can damage cells. This oxidative stress, combined with inflammation, damages the small blood vessels that supply the nerves (vasa nervorum), reducing their ability to get oxygen and nutrients. This can lead to nerve ischemia (lack of oxygen) and further damage.

Glycation of proteins (advanced glycation end products): High blood glucose levels also lead to the formation of advanced glycation end products (AGEs), which are harmful compounds formed when glucose reacts with proteins. AGEs accumulate in nerve tissues, and their presence disrupts normal cellular function, leading to nerve injury and impairing the ability of nerves to regenerate.

Demyelination and axonal damage: The combination of these effects leads to changes in the structure and function of peripheral nerves. Nerve fibers, especially the myelin sheath that insulates nerve axons, can become damaged. Myelin loss leads to slower nerve conduction and even complete nerve failure in severe cases. Axonal degeneration can also occur, which further impairs the nerve’s ability to transmit signals effectively.

Disrupted blood flow: High glucose levels also promote atherosclerosis, or the hardening of the arteries, which can lead to reduced blood flow to peripheral nerves. This ischemic condition can cause nerve damage and eventually lead to peripheral neuropathy [22-24].

Vitamin E and its role in diabetes mellitus and its complications

Vitamin E, a group of eight lipophilic compounds (tocopherols and tocotrienols), is recognized for its antioxidant, anti-inflammatory, and immunomodulatory functions. Among these, α-tocopherol is the most biologically active in humans. Its ability to scavenge free radicals and interrupt lipid peroxidation pathways has drawn attention for potential roles in reducing the oxidative stress burden associated with diabetes and its vascular complications. However, despite mechanistic plausibility, epidemiological and intervention studies have shown conflicting results, with variations possibly attributable to differences in metabolism, dosage, bioavailability, and gut microbiota interactions [25].

Vitamin E and Diabetic Retinopathy

Diabetic retinopathy (DR) is a leading cause of preventable blindness, particularly in younger adults with long-standing diabetes. It is strongly driven by oxidative stress induced by chronic hyperglycemia, which damages retinal endothelial cells and microvasculature. Experimental studies have demonstrated that vitamin E supplementation, especially α-tocopherol, may reduce oxidative retinal damage, improve retinal blood flow, and prevent capillary leakage [26]. Although these findings are encouraging, evidence from large-scale human clinical trials remains limited. Small-scale studies suggest improved endothelial function [27] and retinal circulation, highlighting vitamin E as a possible adjunct in DR management; however, more robust RCTs are required.

Vitamin E and Diabetic Kidney Disease

Diabetic kidney disease (DKD), a major contributor to end-stage renal disease, is characterized by chronic inflammation, hyperglycemia-induced oxidative stress, and endothelial dysfunction. Vitamin E supplementation has been studied for its potential renoprotective effects. Some studies report improved renal parameters (e.g., reduced proteinuria, better endothelial function), while others show neutral or inconclusive results [28]. Meta-analyses indicate mixed benefits on cardiovascular outcomes in diabetic populations, suggesting that while vitamin E may influence oxidative and inflammatory pathways, its impact on long-term renal outcomes remains uncertain. Hence, the evidence for cardiovascular benefits in diabetic populations remains inconsistent, indicating that the effects on renal and cardiovascular outcomes should be interpreted separately.

Antioxidant Role in Diabesity

The coexistence of obesity and diabetes (diabesity) accelerates oxidative stress, aggravating vascular injury and complications. Vitamin E, often in synergy with other antioxidants such as polyphenols and vitamin C, may preserve endothelial function, reduce systemic inflammation, and delay vascular complications [29]. Observational data point towards protective effects of antioxidant-rich diets, but the specific contribution of vitamin E alone is not fully clarified.

Large trials, including HOPE [30], HOPE-TOO [31], and PPP [32], found no significant cardiovascular benefit from 400 IU/day vitamin E supplementation in diabetes. Although mechanistic effects on oxidative stress exist, high-dose supplementation has not shown clear clinical advantage over dietary intake.

Food sources of vitamin E include oils (such as wheat germ oil, sunflower oil, safflower oil), nuts and seeds (such as almonds, hazelnuts, peanuts, peanut butter), fish (such as abalone, trout, salmon), and vegetables and fruits (such as red sweet pepper, turnip greens, butternut squash, avocado, mango, kiwi).

Selenium and its role in diabetes mellitus and its complications

Selenium is an essential trace element incorporated into more than 25 selenoproteins, including GPx and selenoprotein P, which play critical roles in oxidative stress regulation, redox balance, and insulin signaling. Adequate selenium intake supports pancreatic β-cell protection, insulin biosynthesis, and glycemic control. However, a dual relationship exists: while deficiency is associated with impaired glucose metabolism, excess selenium intake has been linked to insulin resistance and diabetes risk [33,34].

Evidence in Diabetes

Observational studies suggest that low selenium intake (≤45 μg/day) is associated with poorer glycemic control and elevated HbA1c levels, particularly in young adults with normal-weight obesity [35]. This indicates selenium deficiency as a potential early risk factor for glycemic dysregulation. Mechanistically, selenoproteins regulate insulin secretion and action, though imbalances such as overexpression of selenoprotein P may paradoxically impair insulin sensitivity and contribute to type 2 diabetes pathogenesis [36]. The study by Stranges et al. [37] showed increased type 2 diabetes risk with 200 µg/day selenium supplementation, suggesting a U-shaped relationship between selenium status and metabolic health. While deficiency impairs antioxidant defense, excess may promote insulin resistance, underscoring the need for balanced, population-specific intake.

Selenium in Wound Healing

Chronic wounds, particularly diabetic foot ulcers (DFUs), remain a major clinical challenge due to oxidative stress, impaired angiogenesis, and chronic low-grade inflammation. Selenoproteins (GPx, selenoprotein S, selenoprotein P) mitigate oxidative burden and support tissue regeneration [38]. In animal models, selenium nanoparticles, especially in combination with platelet-rich plasma, have been shown to accelerate wound closure, reduce necrosis, and enhance granulation tissue formation [39]. Most evidence for selenium’s role in diabetic wound healing comes from preclinical and animal studies showing antioxidant, anti-inflammatory, and tissue-regenerative effects. However, human clinical data remain scarce and largely exploratory. Hence, these findings should be interpreted cautiously until confirmed by robust, well-designed clinical trials.

Food sources of selenium includes nuts and seeds (such as Brazil nuts [richest source], sunflower seeds, cashews, chia seeds), seafood (such as tuna, halibut, shrimp, sardines, oysters, salmon), meat and poultry (such as turkey, chicken, beef, eggs), whole grains and legumes (such as brown rice, wheat bread, oats, lentils, beans), and vegetables and dairy (such as spinach, broccoli, garlic, milk, yogurt).

Vitamin C and its role in diabetes mellitus and its complications

Vitamin C (ascorbic acid) is a water-soluble antioxidant with diverse biological roles, including collagen synthesis, endothelial protection, immunity, and free radical scavenging. In diabetes, hyperglycemia impairs vitamin C uptake into cells by competing with glucose for transport via GLUT transporters, leading to functional deficiency even when dietary intake is adequate.

Evidence in Glycemic Control

Supplementation with vitamin C (500-1000 mg/day) has been associated with modest improvements in glycemic indices (fasting glucose, HbA1c) and blood pressure regulation [40]. It may also enhance antioxidant enzyme activity and reduce systemic inflammation. However, most available studies are small-scale, short-duration trials, with low evidence quality, and large RCTs are warranted.

Vitamin C and Diabetic Complications

Cataracts: Oxidative damage contributes to cataractogenesis, particularly in diabetes. Vitamin C may delay cataract progression and support lens health, though results remain inconsistent across clinical studies [41].

Diabetic ketoacidosis (DKA): Preliminary findings suggest that vitamin C supplementation could reduce oxidative stress and inflammation in DKA, but its therapeutic role in acute management remains underexplored [42].

Immune function: Vitamin C is essential for leukocyte activity, epithelial barrier maintenance, and microbial killing. Deficiency increases infection susceptibility, an important consideration in diabetic wound management [43].

Vitamin C and DFUs

Low serum vitamin C levels are linked with delayed wound healing, poor glycemic control, and altered skin microbiota in patients with DFUs [44]. Although supplementation may aid wound healing, evidence is still emerging, and further controlled studies are required.

The Physicians’ Health Study II [45] found no reduction in cardiovascular or diabetes outcomes with combined vitamin E and C supplementation, highlighting limited clinical translation and supporting a balanced, food-based antioxidant approach.

Food sources of vitamin C include fruits (such as amla, guava, orange, lemon, papaya, pineapple, strawberries, mango, kiwi), vegetables (such as bell peppers, broccoli, cauliflower, cabbage, tomato, green peas), and leafy greens (such as spinach, fenugreek, moringa, amaranth, coriander, curry leaves).

The recommended intakes of all three nutrients are as follows: vitamin E 10 mg/day (as α-tocopherol), selenium 55 µg/day, and vitamin C 45 mg/day [46].

Strengths and Limitations

This narrative review provides an updated synthesis of 25 years of evidence on the roles of vitamin E, vitamin C, and selenium in diabetes and its vascular and neuropathic complications, integrating findings from recent RCTs and mechanistic studies. The inclusion of diverse study designs and populations enhances the breadth of understanding regarding antioxidant-diabetes interactions. However, as a narrative rather than a systematic review, it focuses on descriptive synthesis and may not fully reconcile inconsistencies or weigh evidence based on study quality or sample size. Variations in dosage, intervention duration, and baseline antioxidant status likely contributed to the mixed results reported across studies. While larger and well-conducted RCTs were given contextual emphasis, formal risk-of-bias assessment and evidence grading were beyond the intended scope. These limitations highlight the need for future systematic reviews and rigorously designed trials to strengthen the evidence base for antioxidant supplementation in diabetes management.

The description of the nutrient and its relevance to diabetes and its complications in mentioned in Table 1.

**Table 1:** Nutritional antioxidants (vitamin E, selenium, and vitamin C) in diabetes and its complications: evidence from recent studies This table summarizes key studies on the roles of vitamin E, selenium, and vitamin C in diabetes and its complications. It outlines study objectives, findings, and their relevance to diabetes management and related complications. While vitamin E shows mixed but promising effects on vascular, renal, and retinal outcomes, selenium demonstrates a dual role—protective at adequate intake but potentially harmful in excess—highlighting its impact on insulin signaling and wound healing. Vitamin C emerges as a supportive nutrient for glycemic control, vascular health, and diabetic complications such as cataracts, ketoacidosis, and foot ulcers, though evidence quality and consistency vary. Together, these studies emphasize both the therapeutic potential and limitations of micronutrient supplementation in diabetes care. DKA, diabetic ketoacidosis; DR, diabetic retinopathy; GPx, glutathione peroxidase; PRP, platelet-rich plasma; SeNPs, selenium nanoparticles

Study (Authors, Year)	Nutrient	Objective	Key Findings	Relevance to Diabetes/Complications
Ciarcià et al., 2022 [25]	Vitamin E	Review metabolism of vitamin E, gut microbiota interaction, and its role in non-communicable diseases	Vitamin E has antioxidant and anti-inflammatory effects, but results on chronic disease prevention (e.g., diabetes, cancer) are inconsistent	Highlights conflicting evidence on vitamin E’s role in diabetes and other diseases, suggesting metabolism and microbiota as factors
Asbaghi et al., 2023 [26]	Vitamin E	Explore oxidative stress mechanisms in DR and antioxidant therapy	Oxidative stress drives DR; antioxidants such as vitamin E show promise in animal studies, but human trial data are limited	Suggests vitamin E as a potential therapy for DR, a major diabetes complication, though more clinical evidence is needed
Baburao Jain et al., 2012 [27]	Vitamin E	Investigate vitamin E’s role in preventing diabetic complications	Vitamin E deficiency is common in diabetes; supplementation improves endothelial function, retinal blood flow, and renal function	Indicates vitamin E may mitigate vascular complications in diabetes
Di Vincenzo et al., 2019 [28]	Vitamin E	Review vitamin E supplementation in diabetic nephropathy	Mixed results on cardiovascular outcomes; some studies show renal parameter improvements with supplementation	Suggests potential benefits of vitamin E in diabetic kidney disease, but evidence remains unclear
Dal and Sigrist, 2016 [29]	Vitamin E	Examine oxidative stress, endothelial dysfunction, and antioxidant foods in diabesity complications	Vitamin E (tocopherol) and other antioxidants may reduce oxidative stress and vascular complications	Links vitamin E to prevention of diabetes-related vascular issues via dietary intake
Fontenelle et al., 2018 [33]	Selenium	Update on selenium’s role in insulin resistance mechanisms	Adequate selenium aids insulin action, but excess may contribute to insulin resistance and diabetes	Indicates a dual role of selenium in glycemic control, relevant to diabetes prevention and management
Santos et al., 2021 [35]	Selenium	Evaluate the relationship between dietary selenium intake and glycemic control in young adults with normal-weight obesity	Low selenium intake linked to higher HbA1c; adequate intake may prevent glycemic disturbances	Suggests selenium deficiency as a risk factor for early glycemic issues in at-risk populations
Casanova and Monleon, 2015 [36]	Selenium	Review selenium’s role in type 2 diabetes and insulin pathways	Selenoproteins (e.g., GPx, selenoprotein P) influence insulin signaling and secretion	Highlights selenium’s complex role in type 2 diabetes pathogenesis
Hariharan and Dharmaraj, 2020 [38]	Selenium	Explore selenium/selenoproteins in wound healing	Selenoproteins (e.g., GPx) reduce oxidative stress and aid wound healing	Relevant to diabetic wound healing, a key complication, via antioxidant mechanisms
Karas et al., 2024 [30]	Selenium	Test SeNPs and PRP in diabetic wound healing in mice	SeNPs and PRP synergistically enhance wound healing in diabetic mice, reducing amputation risk	Promising therapy for diabetic foot ulcers, addressing a severe diabetes complication
Mason et al., 2023 [40]	Vitamin C	Review vitamin C supplementation effects and mechanisms in diabetes management	Vitamin C improves glycemic control and blood pressure; promising for diabetic foot ulcers, but evidence quality is low	Suggests vitamin C as a safe, effective adjunct for diabetes management, with gaps in complication-focused research
Lim et al., 2020 [41]	Vitamin C	Investigate vitamin C as an anti-cataract therapy in aging and diabetic populations	Mixed results on cataract prevention; potential in delaying cataracts post-vitrectomy	Relevant to diabetic cataracts, though evidence is inconsistent
Casillas et al., 2018 [42]	Vitamin C	Evaluate vitamin C’s role in treating DKA	Vitamin C’s antioxidant properties may reduce oxidative stress in DKA, but data are preliminary	Explores vitamin C as a potential therapy for an acute diabetes complication
Carr and Maggini, 2017 [43]	Vitamin C	Review vitamin C’s immune functions and supplementation effects	Vitamin C supports immunity, reduces infection risk; higher doses needed during infections	Indirectly relevant to diabetes via infection prevention, which complicates glycemic control
Tong et al., 2022 [44]	Vitamin C	Assess correlations between vitamin C, HbA1c, and microbial communities in diabetic foot ulcers	Low vitamin C levels linked to poor glycemic control and altered skin microbiomes in DFUs	Suggests vitamin C deficiency as a factor in non-healing diabetic foot ulcers

## Conclusions

Vitamin E, selenium, and vitamin C show potential in mitigating oxidative stress and managing diabetes and its complications, though current clinical evidence remains limited and inconsistent. Vitamin E may help reduce the risks of retinopathy and nephropathy, selenium supports insulin signalling and wound healing but may be harmful in excess, and vitamin C contributes to glycemic control, immunity, and wound healing, though findings require further validation. While mechanistic and experimental studies provide strong biological plausibility, the quality and consistency of human trials remain insufficient to support firm recommendations for supplementation beyond maintaining adequate dietary intake. Excessive intake is uncommon in India; however, global recommendations by the WHO/FAO vary according to dietary patterns and population needs. A balanced diet rich in about 500 g of fruits and vegetables, whole grains, legumes, nuts, and low-fat dairy continues to be universally recommended to promote metabolic health and reduce the burden of chronic diseases and their complications.

## References

[1] (2025). Diabetes: fact sheet. https://www.who.int/news-room/fact-sheets/detail/diabetes.

[2] (2014). Diagnosis and classification of diabetes mellitus. Diabetes Care.

[3] Uzun F, Fındık H, Kaim M, Yıldırım M (2024). The relationship between hand abnormalities and diabetic retinopathy in patients with type 2 diabetes mellitus. J Clin Med.

[4] Giacco F, Brownlee M (2010). Oxidative stress and diabetic complications. Circ Res.

[5] Kozlov AV, Javadov S, Sommer N (2024). Cellular ROS and antioxidants: physiological and pathological role. Antioxidants (Basel).

[6] Laxmaiah A, Rao NM, Arlappa N (2022). SARS-CoV-2 seroprevalence in the city of Hyderabad, India in early 2021. IJID Reg.

[7] Oyenihi AB, Ayeleso AO, Mukwevho E, Masola B (2015). Antioxidant strategies in the management of diabetic neuropathy. Biomed Res Int.

[8] Valko M, Leibfritz D, Moncol J, Cronin MT, Mazur M, Telser J (2007). Free radicals and antioxidants in normal physiological functions and human disease. Int J Biochem Cell Biol.

[9] Rahman I, Biswas SK, Kode A (2006). Oxidant and antioxidant balance in the airways and airway diseases. Eur J Pharmacol.

[10] Gupta VK (2012). Bioactive Phytochemicals: Perspectives for Modern Medicine. Bioactive Phytochemicals: Perspectives for Modern Medicine.

[11] Page MJ, McKenzie JE, Bossuyt PM (2021). The PRISMA 2020 statement: an updated guideline for reporting systematic reviews. BMJ.

[12] Rochette L, Zeller M, Cottin Y, Vergely C (2014). Diabetes, oxidative stress and therapeutic strategies. Biochim Biophys Acta.

[13] Khan M, Ali S, Al Azzawi TN, Saqib S, Ullah F, Ayaz A, Zaman W (2023). The key roles of ROS and RNS as a signaling molecule in plant-microbe interactions. Antioxidants (Basel).

[14] Tuell DS, Los EA, Ford GA, Stone WL (2023). The role of natural antioxidant products that optimize redox status in the prevention and management of type 2 diabetes. Antioxidants (Basel).

[15] Weinberg Sibony R, Segev O, Dor S, Raz I (2024). Overview of oxidative stress and inflammation in diabetes. J Diabetes.

[16] Yagihashi S, Mizukami H, Sugimoto K (2011). Mechanism of diabetic neuropathy: where are we now and where to go?. J Diabetes Investig.

[17] Kihara M, Weerasuriya A, Low PA (1991). Endoneurial blood flow in rat sciatic nerve during development. J Physiol.

[18] Sima AA, Nathaniel V, Prashar A, Bril V, Greene DA (1991). Endoneurial microvessels in human diabetic neuropathy. Endothelial cell dysjunction and lack of treatment effect by aldose reductase inhibitor. Diabetes.

[19] Malik RA, Tesfaye S, Newrick PG (2005). Sural nerve pathology in diabetic patients with minimal but progressive neuropathy. Diabetologia.

[20] Thrainsdottir S, Malik RA, Dahlin LB (2003). Endoneurial capillary abnormalities presage deterioration of glucose tolerance and accompany peripheral neuropathy in man. Diabetes.

[21] Obrosova IG (2009). Diabetes and the peripheral nerve. Biochim Biophys Acta.

[22] Hotta N, Kawamori R, Atsumi Y (2008). Stratified analyses for selecting appropriate target patients with diabetic peripheral neuropathy for long-term treatment with an aldose reductase inhibitor, epalrestat. Diabet Med.

[23] Busik JV, Tikhonenko M, Bhatwadekar A (2009). Diabetic retinopathy is associated with bone marrow neuropathy and a depressed peripheral clock. J Exp Med.

[24] Li H, Horke S, Förstermann U (2014). Vascular oxidative stress, nitric oxide and atherosclerosis. Atherosclerosis.

[25] Ciarcià G, Bianchi S, Tomasello B (2022). Vitamin E and non-communicable diseases: a review. Biomedicines.

[26] Asbaghi O, Nazarian B, Yousefi M, Anjom-Shoae J, Rasekhi H, Sadeghi O (2023). Effect of vitamin E intake on glycemic control and insulin resistance in diabetic patients: an updated systematic review and meta-analysis of randomized controlled trials. Nutr J.

[27] Baburao Jain A, Anand Jain V (2012). Vitamin E, its beneficial role in diabetes mellitus (DM) and its complications. J Clin Diagn Res.

[28] Di Vincenzo A, Tana C, El Hadi H, Pagano C, Vettor R, Rossato M (2019). Antioxidant, anti-inflammatory, and metabolic properties of tocopherols and tocotrienols: clinical implications for vitamin E supplementation in diabetic kidney disease. Int J Mol Sci.

[29] Dal S, Sigrist S (2016). The protective effect of antioxidant consumption on diabetes and vascular complications. Diseases.

[30] Yusuf S, Dagenais G, Pogue J, Bosch J, Sleight P (2000). Vitamin E supplementation and cardiovascular events in high-risk patients. N Engl J Med.

[31] Lonn E, Bosch J, Yusuf S (2005). Effects of long-term vitamin E supplementation on cardiovascular events and cancer: a randomized controlled trial. JAMA.

[32] Roncaglioni MC (2001). Low-dose aspirin and vitamin E in people at cardiovascular risk: a randomised trial in general Practice. Lancet.

[33] Fontenelle LC, Fechine FV, Neto JMC (2018). Role of selenium in insulin resistance. Braz J Pharm Sci.

[34] Ouyang J, Cai Y, Song Y, Gao Z, Bai R, Wang A (2022). Potential benefits of selenium supplementation in reducing insulin resistance in patients with cardiometabolic diseases: a systematic review and meta-analysis. Nutrients.

[35] Santos AC, Passos AF, Holzbach LC, Cominetti C (2021). Selenium intake and glycemic control in young adults with normal-weight obesity syndrome. Front Nutr.

[36] Casanova P, Monleon D (2023). Role of selenium in type 2 diabetes, insulin resistance and insulin secretion. World J Diabetes.

[37] Stranges S, Marshall JR, Natarajan R (2007). Effects of long-term selenium supplementation on the incidence of type 2 diabetes: a randomized trial. Ann Intern Med.

[38] Hariharan S, Dharmaraj S (2020). Selenium and selenoproteins: it's role in regulation of inflammation. Inflammopharmacology.

[39] Karas RA, Alexeree S, Elsayed H, Attia YA (2024). Assessment of wound healing activity in diabetic mice treated with a novel therapeutic combination of selenium nanoparticles and platelets rich plasma. Sci Rep.

[40] Mason SA, Parker L, van der Pligt P, Wadley GD (2023). Vitamin C supplementation for diabetes management: a comprehensive narrative review. Free Radic Biol Med.

[41] Lim JC, Caballero Arredondo M, Braakhuis AJ, Donaldson PJ (2020). Vitamin C and the lens: new insights into delaying the onset of cataract. Nutrients.

[42] Casillas S, Pomerantz A, Surani S, Varon J (2018). Role of vitamin C in diabetic ketoacidosis: is it ready for prime time?. World J Diabetes.

[43] Carr AC, Maggini S (2017). Vitamin C and immune function. Nutrients.

[44] Tong KP, Green SJ, Ortiz J, Wu SC (2022). Association between hemoglobin A1c, Vitamin C, and microbiome in diabetic foot ulcers and intact skin: A cross-sectional study. Health Sci Rep.

[45] Sesso HD, Buring JE, Christen WG (2008). Vitamins E and C in the prevention of cardiovascular disease in men: the Physicians' Health Study II randomized controlled trial. JAMA.

[46] Expert consultation (2025). Human vitamin and mineral requirements. World Health Organization & Food and Agriculture Organization of the United Nations. (2004). Human vitamin and mineral requirements: Report of a joint FAO/WHO expert consultation, Bangkok, Thailand. 2nd ed. Geneva: World Health Organization..

